# Agronomic improvement using gamma ray induced mutagenesis is associated with changes in phytochemical and phytohormonal profiles in functional rice variety ‘Gathuwan’

**DOI:** 10.1186/s12870-025-07036-1

**Published:** 2025-08-12

**Authors:** Anjali Chauhan, Rahul Checker, Parmeshwar K. Sahu, Raviraj Singh Patel, Samrath Baghel, Deepak Sharma, Deepak Sharma, Bikram K. Das

**Affiliations:** 1 Nuclear Agriculture and Biotechnology Division, Bhabha Atomic Research Centre, Trombay, Mumbai, 400085 India; 2 Radiation Biology & Health Sciences Division, Bio-Science Group, Bhabha Atomic Research Centre, Trombay, Mumbai, 400085 India; 3 Homi Bhabha National Institute, Anushaktinagar, Mumbai, 400094 India; 4 Department of Genetics and Plant Breeding, Indira Gandhi Krishi Vishwavidyalaya, Raipur, 492012 India; 5 RABL College of Agriculture & Research Station, Indira Gandhi Krishi Vishwavidyalaya, Chhuikhadan, 491885 India

**Keywords:** Rice, Gamma rays, Mutation breeding, Metabolomics, Phytochemicals, Phytohormones

## Abstract

**Background:**

Gamma ray induced mutation breeding has emerged as an excellent method for expedited development of improved varieties of rice, a staple food for more than half the world's population. However, the assessment of radiation induced variations are primarily phenotypic in nature. In this direction, evaluation of the metabolic signature of bio-active ingredients, which confer beneficial properties to rice, could be employed as a tool to select varieties which not only retain the health benefits of the parent variety but also exhibit better agronomic traits. The present study was, therefore, aimed at evaluating the metabolomic changes in the mutants of Gathuwan, an indigenous Indian rice with immunomodulatory properties. The mutant varieties were developed through gamma irradiation, and liquid chromatography-tandem mass spectrometry (LC–MS/MS)-based metabolic profiling was performed.

**Results:**

A total of 274 differentially expressed compounds were identified among Gathuwan and four of its mutants (mutant 6, mutant 7, mutant 8 and mutant 12), indicating that gamma irradiation induced stable metabolic alterations. Significant differences were observed in the phytochemical composition of mutants relative to the parent, emphasizing the importance of metabolic screening in functional rice breeding. Cluster analysis and phytochemical profiling revealed that mutant 6 was metabolically closest to the parent variety. Additionally, distinct phytohormonal variations among the mutants were observed which may account for the phenotypic differences in growth and development.

**Conclusions:**

Our study demonstrates that radiation-induced improvement in agronomic traits are accompanied by distinct alterations in phytochemical and phytohormonal profiles in stable rice mutants. These metabolic changes support the functional potential of the mutants and provide insights into the biological mechanisms underlying their traits. Among the mutants, mutant 6 emerges as a promising candidate due to its similarity to the parent in metabolite composition. Therefore, inclusion of metabolomic profiling as a selection criterion offers a powerful tool to identify robust and functionally superior rice varieties.

**Supplementary Information:**

The online version contains supplementary material available at 10.1186/s12870-025-07036-1.

## Background

Rice is consumed by more than half of world’s population for calories and basic nutrition. In 2022–23, worldwide consumption of rice was 520.4 million metric tons, of which a significantly large proportion was consumed by developing nations (https://www.statista.com). Largescale dependency on rice makes it a crucial contributor to the global food security and captivates more emphasis toward sustainable rice production. However, two important aspects which need to be considered for ensuring food security in future are that rice is a crop with vast diversity in agro-morphological traits along with nutritional and sensory qualities. The most widely consumed form of rice is polished rice, wherein the processing stripes off most of the nutritive elements. Numerous studies have shown unpolished rice to be healthier than the polished rice due to retention of bran and germ, the anatomical fractions of rice containing many nutrients and phytochemicals [1–3]. Emerging evidence indicates that among the plethora of existing rice cultivars, the traditional ones which have adapted and stood the test of time over thousands of years, produce biochemicals with beneficial effects on human health and disease conditions [4]. Many traditional rice varieties from countries like India, China, Thailand, Sri-Lanka etc. have been found to exhibit therapeutic effects as diverse as anti-oxidative, anti-allergic, anti-cancer, anti-diabetic, immune-stimulatory, cardioprotective, hepatoprotective etc., supporting activity based ethnic classifications of rice [5–9]. However, the lower yield of traditional varieties is a major drawback for their long-term commercial viability. Poor plant and panicle architecture along with long maturity duration are the factors responsible for the low yield. Hence there is a clear need to identify novel strategies that can be applied to overcome the undesirable traits associated with rice verities having health promoting properties.

In this direction, inducing desired heritable changes in the phenotype of plants using physical mutagenic agents is an effective way to overcome the limitations of rice landraces within comparatively shorter span of time. Although radiation induced mutagenesis is a random process, it has the potential to generate wide combinations of traits providing opportunity to select and propagate the desirable one. Therefore, gamma ray induced mutagenesis has been extensively used for the improvement of yield, harvest time and efforts, nutrient use efficiency, stress tolerance and nutritional and organoleptic qualities in various crops [10–12], thus contributing significantly to food sustainability. Gamma rays induce broad spectrum mutations which manifests as diverse phenotypic, physiological and biochemical changes [13–15]. In crop improvement programs, major emphasis is laid on either phenotypic or physiological alterations. However, gamma rays have also been shown to alter contents of various biochemicals present in these varieties and also affect plant responses to various stresses [16–18]. These biochemical are often implicated in regulating the biological activities of different parts of plants and also known to be responsible for health promoting effects in humans [19–22]. In a study by Kim et al. (2004), metabolic changes led to balanced amino acid composition by enhancing the levels of limiting amino acids (lysine and tryptophan) in rice, along with increased protein content [23]. Kongdin et al. (2023) also showed the effect of gamma rays on phenolic compound accumulation in rice under stress conditions [24]. Taken together, these observations indicate that the mutant varieties may possibly acquire a different metabolic signature as compared to the parent variety due to altered biochemical pathways, particularly in case of plants of nutraceutical worth.

It is well accepted that the metabolome of a plant is an aggregation of all the metabolites (primary and secondary) and is responsible for shaping the morphology and functional properties of the plant. Therefore, in recent years, there has been an increased focus on deciphering the metabolic signature of plant products associated with their biological activity in different organisms. A large number of metabolic investigations are being conducted in rice for diverse purposes such as metabolic profiling, discriminating different rice cultivars, plant interaction with biotic and abiotic factors, stress response and integrated studies to understand functional gene-metabolite relationships [25–30]. Hence, utilizing a metabolomics guided approach can prove to be a valuable tool for the improvement of indigenous rice having medicinal properties.

In the present study, mutation breeding using gamma rays was employed to improve the agronomic traits of Gathuwan rice, a traditional rice variety originally collected from Abhanpur district of Chhattisgarh, India. It is being maintained as a part of India’s largest rice germplasm collection at Indira Gandhi Krishi Vishwavidyalaya (IGKV), Raipur, Chhattisgarh. We have recently showed that Gathuwan brown rice extract exhibits potent immune-suppressive functions, which may be responsible for its healing effects in the patients suffering from inflamed joints (arthritis) [31]. The biological activity of Gathuwan rice was attributable to the enriched metabolites correlating with the functional response [31]. This rice landrace, however, suffers marginalised cultivation mainly because of the tall height of the plant and longer maturity duration. Here, we have used gamma ray-induced mutation breeding to select the mutants with improved agronomic traits and employed metabolic profiling to identify the mutant which is closest to the parent. Our results highlight that metabolic profiling is particularly crucial for improving cultivars with biological properties resulting from interaction of specific components.

## Methods

### Mutant development

For mutagenesis, pure seeds of Gathuwan rice were obtained from Department of Genetics and Plant Breeding, IGKV, Raipur in the year 2019. The seeds (M0) were irradiated with 250 Gy of gamma rays at Nuclear Agriculture & Biotechnology Division (NA&BTD), Bhabha Atomic Research Centre (BARC), Mumbai. A population of 2500 plants was raised in M1 generation during *Rabi* season 2019–20 (Dry season 2019–20) and mother panicle of individual plant was harvested separately and kept in envelopes. M2 generation was raised using panicle-to-row method during *Kharif* season 2020 (Wet season 2020). Single panicle seeds were sown in single line at spacing of 15 cm, while inter-line distance was 20 cm. A thorough screening of approximately 18,000 plants in M2 generation for reduced plant height, early maturity duration and better yielding ability resulted in isolation of 9 mutants (plants with genetically altered phenotypes) with reduced plant height or flowering time or both. These mutants were tested for homozygosity in M3 generation during *Rabi* season 2020–21 (Dry season 2020–21), as the sowing was done using plant-to row method. They were further advanced to M4, M5 and M6 generation for stabilization and agro-morphological evaluation during subsequent seasons. Four homozygous mutant lines in M6, along with the parent, were subjected to metabolomic analysis during *Kharif* season 2022 (Wet season 2022). Comprehensive agronomic data was recorded, with plot replications, for mutants and parent in M6 generation.

### LC–MS/MS profiling of Gathuwan and its mutants

#### Sample preparation

Brown rice samples of Gathuwan and four mutants of Gathuwan, were ground to fine powder in liquid nitrogen. The extraction of metabolites from brown rice powder was carried out using method described by Lau et al. (2015), with few modifications [32]. Briefly, 1 ml of extraction solvent mixture containing water, methanol and acetonitrile in 1:2:2 ratio, was added to the 50 mg of powdered brown rice samples. The samples were vortexed for 15 min at room temperature, followed by sonication (in water bath) for 15 min. The samples were centrifuged at 10,000 rpm for 15 min at 4 °C. Supernatant was collected from each sample, transferred to vials for LC and then subjected to LC–MS/MS analysis.

#### Liquid chromatography with tandem mass spectrometry (LC–MS-MS) analysis

The metabolite enriched extracts were injected into Agilent 1290 infinity II liquid chromatography system with a C18 RRHD Zorbax column (20 × 150 mm, 1.8 μm particle size), coupled with QTRAP 6500 mass spectrometer (ABSciex). The separation of the metabolites was carried out using a 25-min LC (liquid chromatography) method. The mobile phase comprised of solvent A (0.1% formic acid in ultrapure water) and solvent B (0.1% formic acid in 90% acetonitrile) while the flow rate was kept constant at 0.25 ml/min. The gradient used in LC was: 2% B for 1–10 min, 30% B at 10–14 mi, 60% B at 14–18 min, 95% B for 18–21 min and 2% B for 21–25 min. Analyst software version 1.6.3 was used for data acquisition and the Analyst Device Driver for setting the parameters for the analysis. Mass spectrometric data acquisition was carried out with IDA method (Information dependent acquisition) in low mass mode. The IDA method was built using the EMS (enhanced mass spectra) to EPI (enhanced product ion) modes. The top five spectra from the EMS mode were used for analysis in the EPI (MS/MS) mode, using high energy CID i.e. collision induced dissociation. The metabolite data was acquired in both positive and negative polarities at 4500 V and −4500 V respectively, with a probe temperature of 450 °C. The compound parameters were set at a declustering potential (DP) of 100 V, collision energy (CE) of 10 V. The data were acquired for all the samples, in triplicates, along with pooled Quality Control (QC). All the resultant files (wiff format) were analysed for metabolite hits.

#### Analysis for MS2 data

The resultant files obtained from LC–MS/MS were directly uploaded for data processing using MZmine tool. The feature detection was carried out with an m/z tolerance of 0.05 Da. It was followed by chromatogram deconvolution using the noise amplitude algorithm with amplitude of noise as 1.5E2. Isotopes were detected using Isotopic peak grouper with an m/z tolerance of 0.25 Da and retention time tolerance of 0.2 min. Alignment of peaks was performed by Join Aligner with an m/z tolerance of 0.05 Da and a retention time tolerance of 0.5 min. After processing, the data was searched against the PlantCyc (BioCyc) metabolite database using MS2Compound software. The metabolite identification and annotation were carried out with a precursor mass tolerance of 0.05 Da and fragment mass tolerance of 0.5 Da. Adducts that were used for the positive mode were [M + H], [M + Na], [2 M + H] and [M + 2H] while, for the negative mode, [M-H], [2 M-H] and [M-2H] were used. The peak areas were mapped with their respective identifications. MS2 data matrix was subjected to final scrutiny for removal of any duplications.

#### Enrichment, pathway and statistical analyses

Enrichment analysis of the annotated metabolites was carried out using Metaboanalyst 6.0 webserver. MS2 annotations were mapped against the server library. For unmatched compounds names, synonymous names were used from PubChem library. Kyoto Encyclopedia of Genes and Genomes (KEGG) based over-representation (enrichment) analysis of pathways and compound classes was performed for individual samples. Quantitative enrichment analysis was performed for pairwise comparison between each mutant and the parent. Sample data was normalized by median and log transformed for comparative weighted analyses. Statistical analyses, for pair-wise comparison of metabolite expression profiles of mutant and parent, included fold change analysis (FC threshold- 2.0), t-test, features correlation, Principal Component Analysis (PCA), Partial Least Squares-Discriminant Analysis (PLS-DA) and cluster analysis indicating the metabolite expression profiles of the samples. ANOVA was performed for simultaneous comparison of parent and all four mutants and dendrogram was built based on comparative results.

## Results

### Development of Gathuwan rice mutants

In M2 generation, where the initial mutant screening was conducted, Gathuwan parent attained a height of 150 cm, reached 50% flowering in 110 days and matured in 145 days. Following screening, 9 mutant plants exhibiting either reduced plant height (up to 30 cm shorter), earlier maturity (up to 15 days earlier), or both, were selected. In the M3 generation, five segregating lines were discarded, and four homozygous mutant lines were advanced through M4 to M6 generations for stabilization and agro-morphological evaluation. Lines designated as mutant 6, mutant 7, mutant 8 and mutant 12 expressed stable expression of the traits in M3-M6 generations. In the M2 generation, plant height of these mutants ranged from 118 to 135 cm, with 50% flowering occurring between 95 and 98 days (Table 1). In the M6 generation, plant height ranged from 116 to 131 cm, and 50% flowering occurred between 91 and 102 days (Table 2). Among these, mutant 6 emerged as the most promising line, exhibiting consistent reduction in plant height and earlier flowering, along with significantly higher yield per plant compared to both the parent and other mutant lines. This increase in yield was primarily attributed to enhanced spikelet fertility. These findings highlight mutant 6 as a strong candidate for further evaluation and potential use in breeding programs with the aim of improving the yield and maturity in rice.

**Table 1:** Agronomic data of Gathuwan and its mutants isolated in M2 generation

Name of genotype	Days to 50% Flowering (days)	Days to Maturity (days)	Plant Height (cm)	Panicle Length (cm)	Flag Leaf Length (cm)	Flag Leaf Width (cm)	Effective Tillers/Plant	Grain Yield/Plant (g)
Gathuwan Parent	110	145	150	22	17.5	1.5	10	19
Gathuwan Mutant-6	95	125	118	21	16.5	1.5	12	26
Gathuwan Mutant-7	97	127	121	22	17.5	1.5	9	23
Gathuwan Mutant-8	98	128	128	22	17.3	1.5	9	21
Gathuwan Mutant-12	95	125	135	23	16.8	1.6	8	20

**Table 2:** Comprehensive agronomic data recorded for Gathuwan and its mutants in M6 generation. (*n* = 10)

Name of genotype	Days to 50% flowering	Plant Height (cm)	Panicle Length (cm)	Total Tillers/plant	Productive Tillers/Plant	Flag Leaf Length (cm)	Flag Leaf Width (cm)	Sterile Spikelets/Panicle	Fertile Spikelets/Panicle	Total spikelets/Panicle	Spikelet fertility %	Grain Yield per plant (g)
Gathuwan (Parent)	111 ± 1.41	152.25 ± 6.01	21.84 ± 1.18	8.50 ± 0.71	8.50 ± 0.71	21.50 ± 0.71	1.51 ± 0.02	25.50 ± 3.54	88 ± 2.83	113.50 ± 0.71	77.54 ± 2.98	18.50 ± 0.71
Mutant 6	94 ± 1.41	126.75 ± 3.18	23.5 ± 0.71	10.5 ± 0.71	10 ± 0.00	21.06 ± 0.86	1.49 ± 0.08	16 ± 2.83	83 ± 2.83	99 ± 0.00	83.84 ± 2.86	23.5 ± 0.71
Mutant 7	98 ± 1.41	116.5 ± 2.12	21.5 ± 0.71	8.5 ± 0.71	8 ±.00	20 ± 1.41	1.44 ± 0.06	25.5 ± 2.12	74.5 ± 0.71	100 ± 1.41	74.51 ± 1.76	18.5 ± 0.71
Mutant 8	102 ± 0.00	126 ± 1.41	20.5 ± 0.71	8.5 ± 0.71	8.5 ± 0.71	20.5 ± 0.71	1.42 ± 0.03	24 ± 2.83	78.5 ± 0.71	102.5 ± 3.54	76.62 ± 1.95	21.5 ± 0.71
Mutant 12	91 ± 1.41	131 ± 1.41	21.5 ± 2.12	8 ± 1.41	8 ± 1.41	21.5 ± 0.71	1.49 ± 0.03	26.5 ± 2.12	84 ± 2.83	110.5 ± 4.95	76.04 ± 0.85	19.5 ± 0.71

(The phenotypic data represents average values of plot replicates. In each replication, average values of measurements from five plants were considered)

### Metabolite analysis

**Fig. 1:**
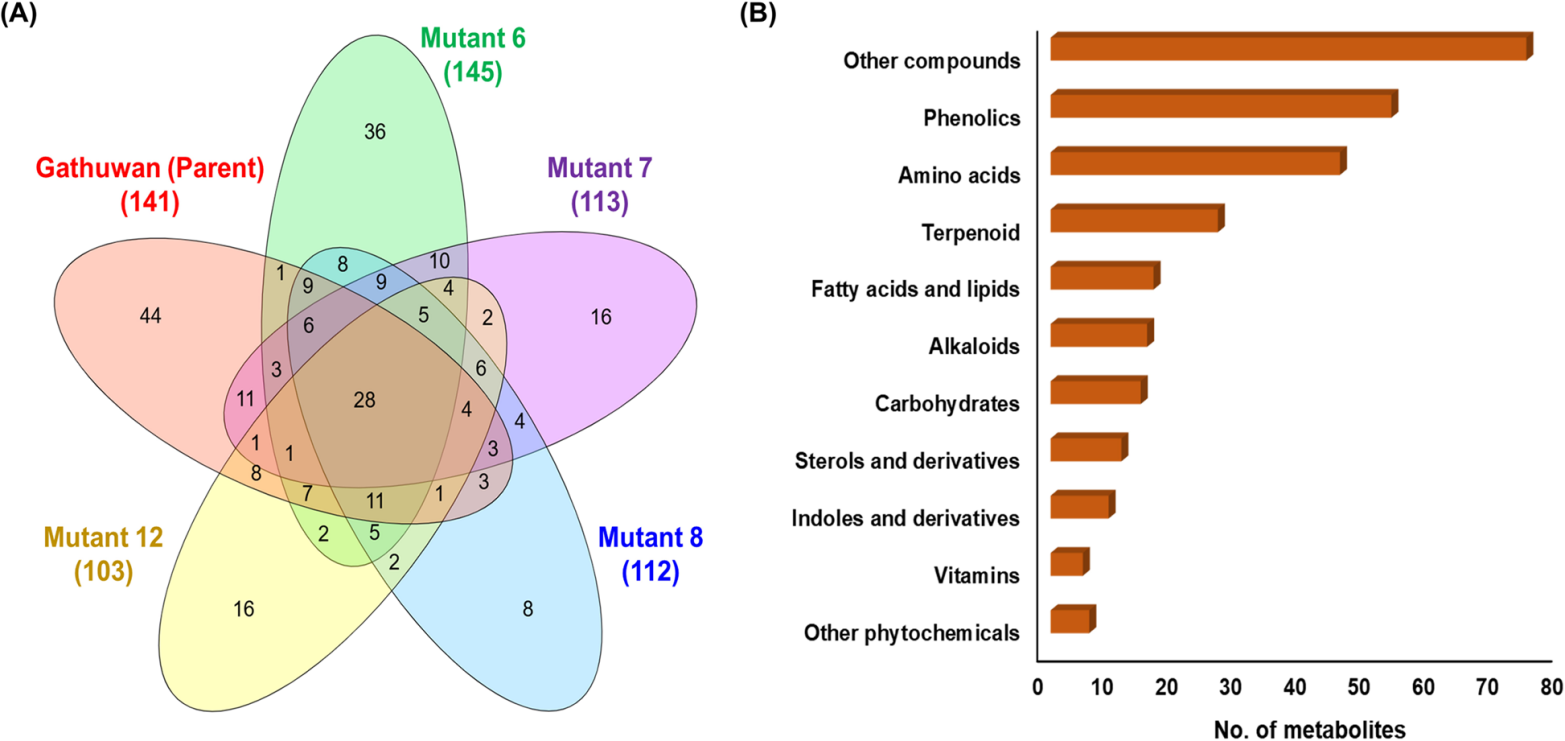
Distribution of metabolites detected using LC–MS/MS among samples studied (**A**) Vein diagram showing common and unique metabolites among Gathuwan and its mutants and (**B**) Bar graph showing number of metabolites corresponding to different compound classes. Other phytochemicals include cyanohydrins, glucosinolates, phytocannabinoids etc. Other compounds include intermediary metabolites involved in the synthesis of final products

A total of 274 compounds corresponding to well annotated MS2 peaks, were used for comparison between parent and mutants. These compounds belonged to various classes, viz., alkaloids, benzenoids, lignans, lipids, nucleosides, phenylpropanoids and polyketides, organic acids, organoheterocyclic, organic nitrogen, organic oxygen and organosulfur compounds. In the metabolic profiling, Gathuwan (parent) brown rice exhibited 141 compounds while brown rice of mutant 6, mutant 7, mutant 8 and mutant 12 respectively showed 145, 113, 112 and 103 compounds, respectively. The number of compounds commonly shared among parent-mutant pair were 66, 57, 65 and 61 respectively. Out of these, 28 metabolites were present in all of the groups whereas 44, 36, 16, 8 and 16 compounds were unique to parent, mutant 6, mutant 7, mutant 8 and mutant 12 respectively (Fig. 1A). mutant 6 had highest percentage (45.5%) of total parental metabolites. In order to study the functional relevance of metabolic changes, the metabolites were classified into primary and secondary metabolite classes as shown in Fig. 1B (S2.1 of Supplementary file S1).

To explore clustering patterns based on differential metabolic features, unsupervised principal component analysis (PCA) was performed to visualize group separation without prior classification. To further identify significant differences among groups, supervised partial least squares discriminant analysis (PLS-DA) was conducted, which maximizes group separation and highlights key discriminant metabolites. Pairwise comparison results from these analyses are discussed in the subsequent sections. Data normalization procedures and sample quality assessments are illustrated in Supplementary Figures S1A–B, while the accuracy, goodness-of-fit (R^2^), and predictive ability (Q^2^) metrics that validate the quality of the multivariate models are presented in Supplementary Figures S2A–D. These steps confirm the robustness and reliability of the statistical analyses performed.

#### Enrichment analysis

**Fig. 2:**
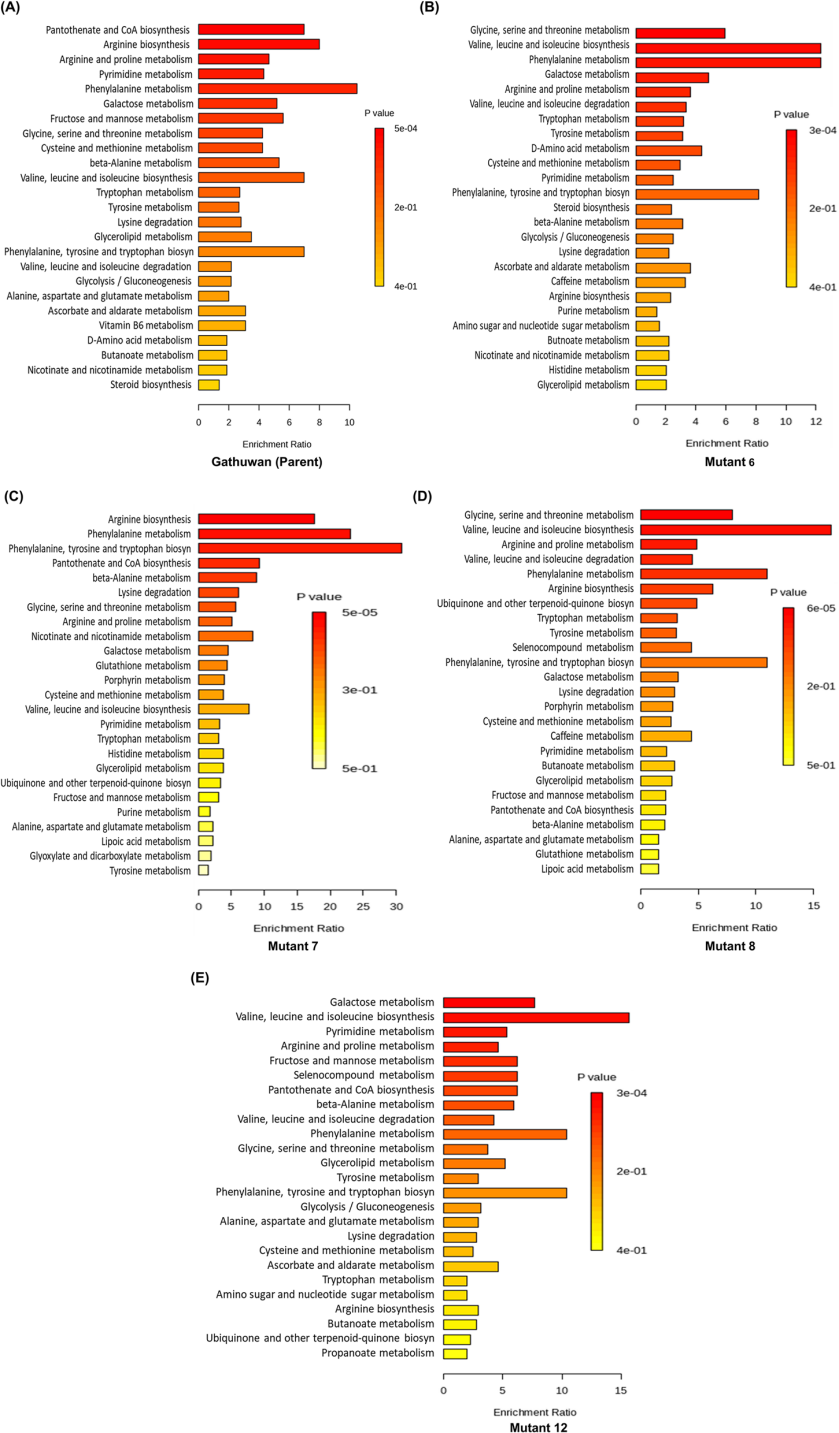
Metaboanalyst 6.0 based pathway enrichment analysis in (**A**) Gathuwan, (**B**) mutant 6, (**C**) mutant 7, (**D**) mutant 8 and (**E**) mutant 12. Top 25 Pathways are arranged on the basis of their significance, which is also indicated by their significance values. Length of each bar represents the enrichment ratio while intensity of colour of the bar indicates their significance

Over-representation analysis of metabolic pathways in Gathuwan parent and mutants showed differences in relative significance and enrichment ratios (Fig. 2A-E). Enrichment of arginine biosynthesis, branched chain amino acid synthesis and Vitamin B6 metabolism pathways in Gathuwan are considered to be related with its immune-suppressive activity [31]. We compared pathway enrichment among mutants with the parent variety and observed that all these three pathways were among top 25 highly enriched pathways in Gathuwan, wherein arginine biosynthesis had highest enrichment ratio and lowest p-value. Among mutants, mutant 6 and 12 had lower while mutant 7 had higher enrichment of arginine biosynthesis. mutant 8 and 12 had highest enrichment of branched chain fatty acid synthesis, followed sequentially by mutant 6, parent and mutant 7. However, enrichment ratios of metabolism of arginine and branched chain amino acids were similar among the samples undertaken. Vitamin B6 metabolism was highly perturbed in mutants as it was not among top 25 enriched pathways in mutants. Since phenylalanine is a substrate for synthesis of many secondary metabolites, synthesis and metabolism of phenylalanine was also compared among the samples. It was observed that phenylalanine metabolism was dominant over its synthesis in parent and mutant 6, with more enrichment in latter. mutant 7 also had higher prevalence of phenylalanine metabolism but it could be accounted to enriched synthesis. The two processes were mutually balancing in mutant 8 and mutant 12.

#### Pathway analysis

**Fig. 3:**
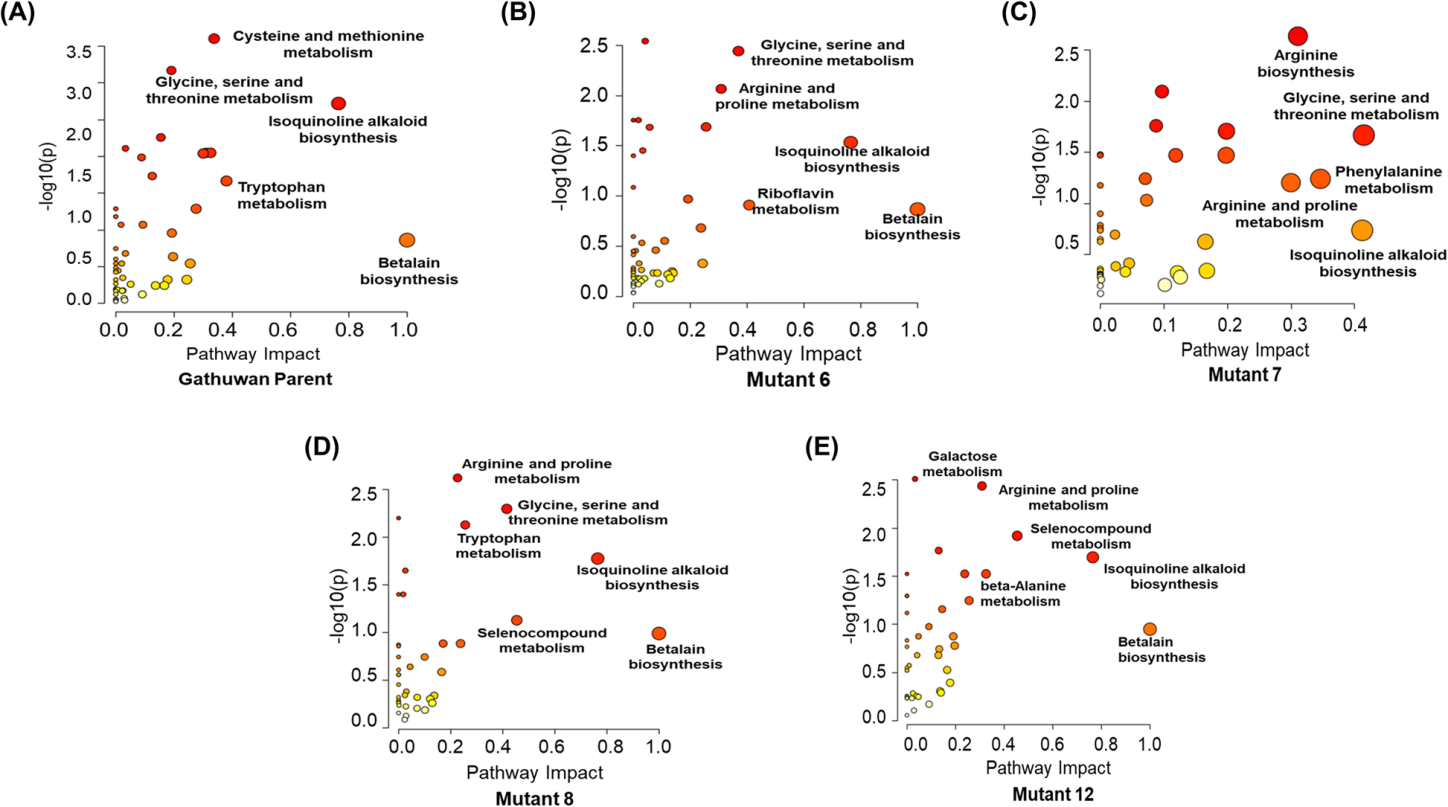
Metabolomic Pathway Analysis (MetPA) to highlight significant impact of pathways, based on topology and impact scores in (**A**) Gathuwan, (**B)** mutant 6, (**C**) mutant 7, (**D**) mutant 8 and (**E**) mutant 12. Pathways are displayed as circles on a plot between pathway impact score (x-axis) and log p-values (y-axis). The size of circle corresponds to impact score, while the intensity of color depends on the *p*-values. Highly impacted pathways with high statistical significance are mentioned in the plots

Pathway impact analysis is a measure to decipher the significant pathways based on abundance and topological importance of metabolites. parent variety had higher impact scores for secondary metabolite synthesis including betalain and isoquinoline alkaloids and tryptophan metabolism. Higher impact of both these secondary metabolite synthesis pathways was also observed among mutant 6, mutant 8 and mutant 12. mutant 8 had higher impact of tryptophan metabolism also among these three mutants. mutant 7 had comparatively higher impact scores but lower impact of pathways indicating a localized metabolic response than pathway alteration **(**Fig. 3A-E**)**.

#### Metabolic diversification among rice mutants

**Fig. 4:**
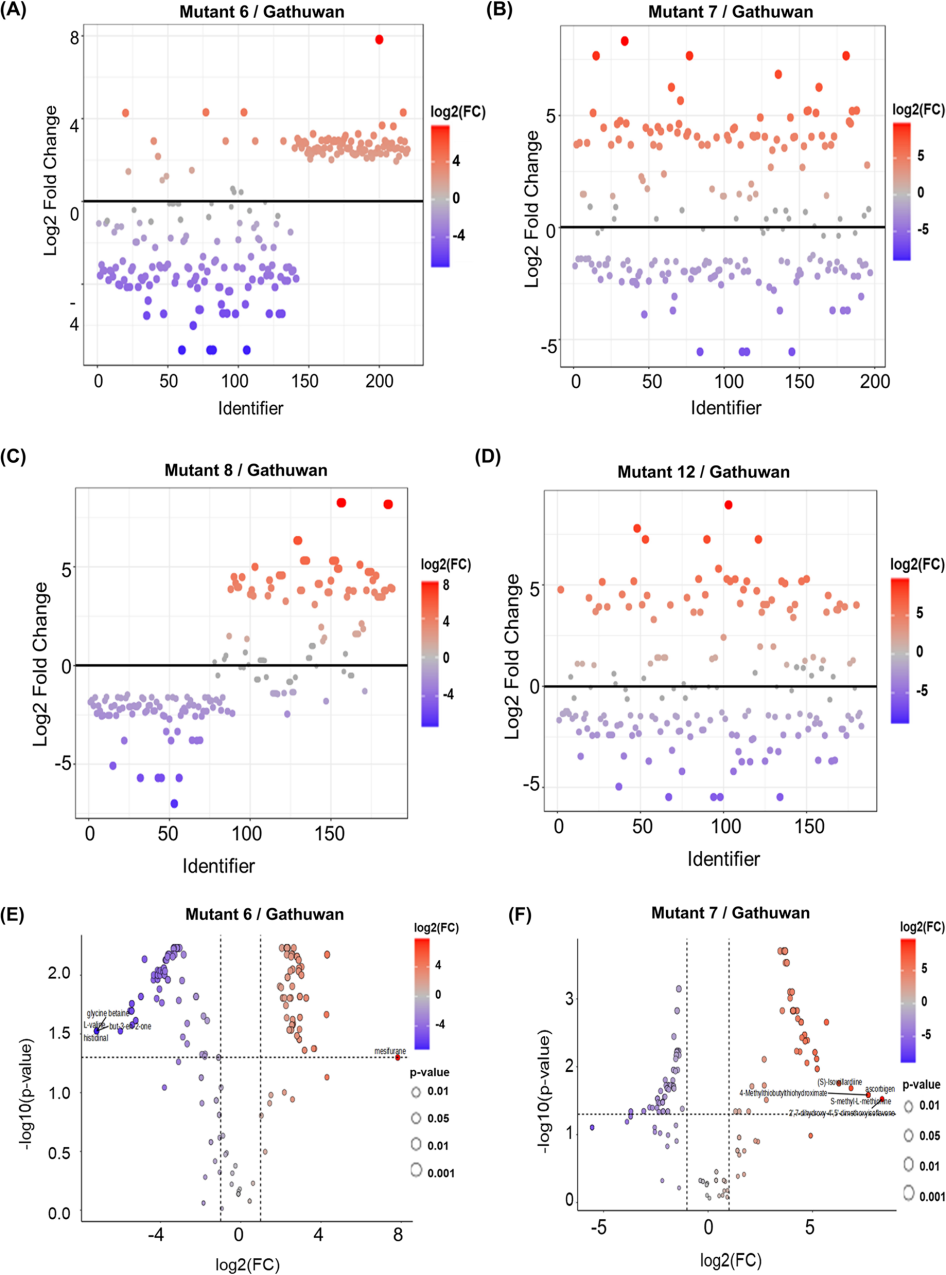
Univariate analysis to identify significantly altered features between Gathuwan and mutants. **A**-**D** Fold change analysis (mutant/parent) to highlight biological significance, and (**E**–**H**) Volcano plot to identify statistically significant features respectively in parent-mutant 6, parent-mutant7, parent-mutant 8 and parent-mutant 8 pairs

For testing the significantly altered features among mutants, fold change analysis (Threshold 2.0) and t-test (FDR adjusted p-value threshold 0.05) were employed, results of which are shown in Fig. 4 A-D and Fig. S3 A-D (Supp. Figures) respectively. Fold change analysis of metabolites in mutants 6, 7, 8, and 12, with respect to the parent, showed the number of upregulated metabolites to be 92, 81, 68, and 68 respectively, while downregulated metabolites numbered 110, 91, 93, and 89 The number of metabolites showing statistically significant differences in mean abundance compared to the parent were 175, 142, 139, and 109 for mutants 6, 7, 8, and 12, respectively (Table S2.2 in Supplementary File S1). The volcano plots shown in Fig. 4 E–H combine fold change and significance to identify biologically meaningful metabolic changes. Across all mutants, significantly downregulated metabolites outnumbered upregulated ones. Among these, mutant 6 had the highest number of upregulated and significant features compared to the parent.

**Fig. 5:**
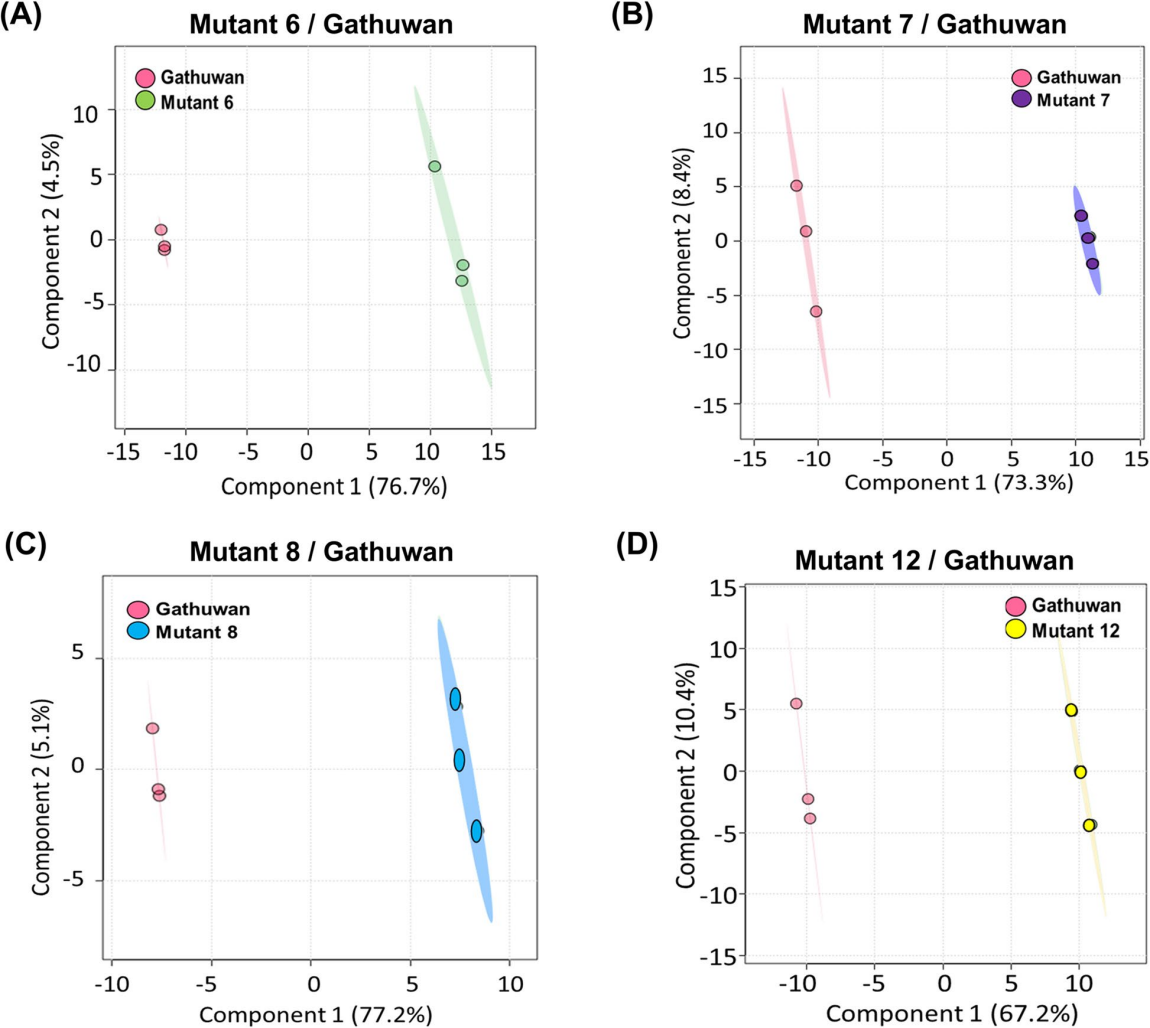
Multivariate analysis for visualization of the difference between Gathuwan and its mutants, in reduced dimensions. Scores plot showing variant grouping of (**A**) parent and mutant 6, (**B**) parent and mutant 7, (**C**) parent and mutant 8 and (**D**) parent and mutant 12 in PLS-DA analysis

**Fig. 6:**
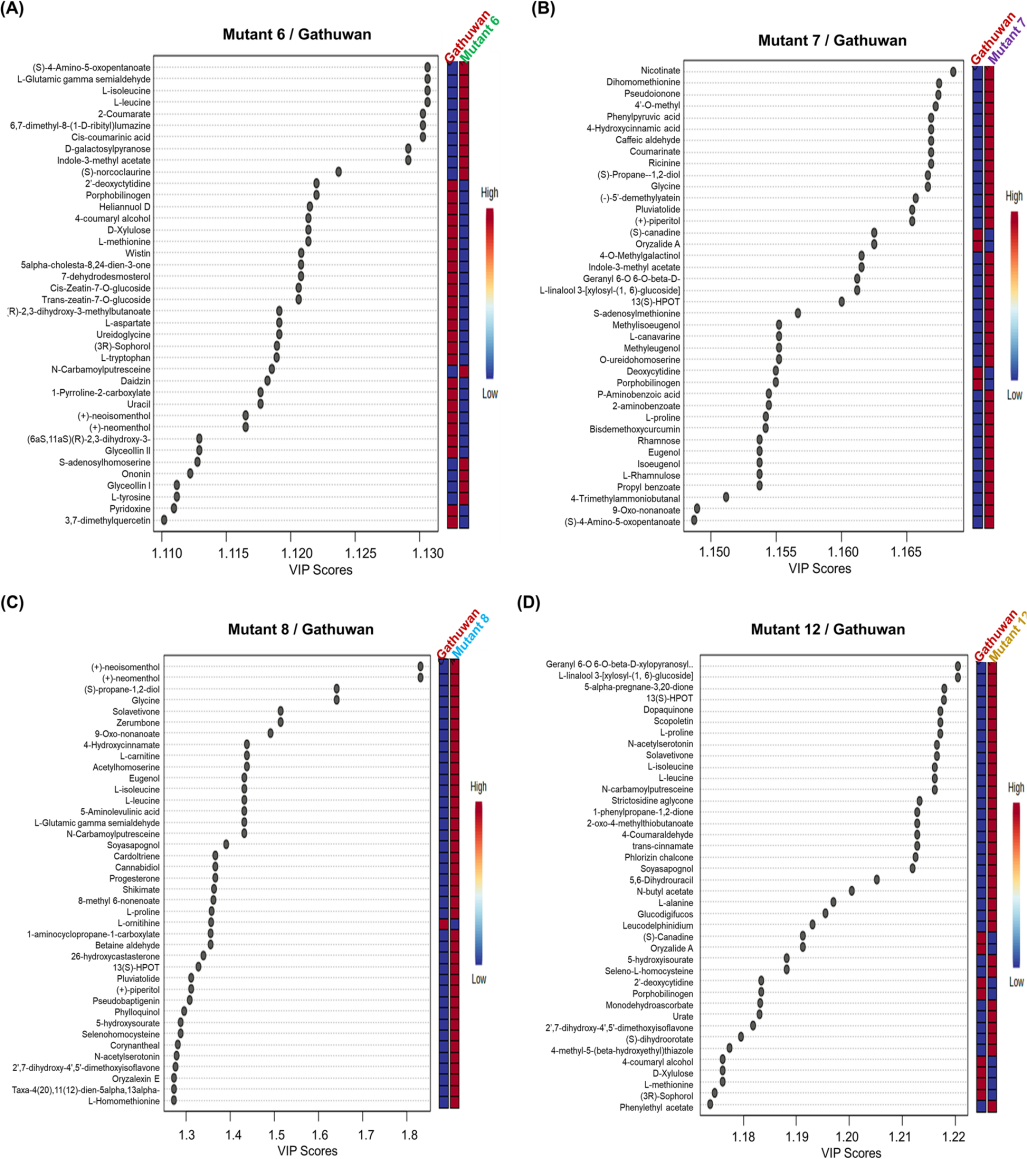
Top 40 important features identified using variable importance in the projection (VIP) scores of PC1 component, respectively in (**A**) parent-mutant 6, (**B**) parent-mutant7, (**C**) parent-mutant 8 and (**D**) parent-mutant 8 pairs

**Fig. 7:**
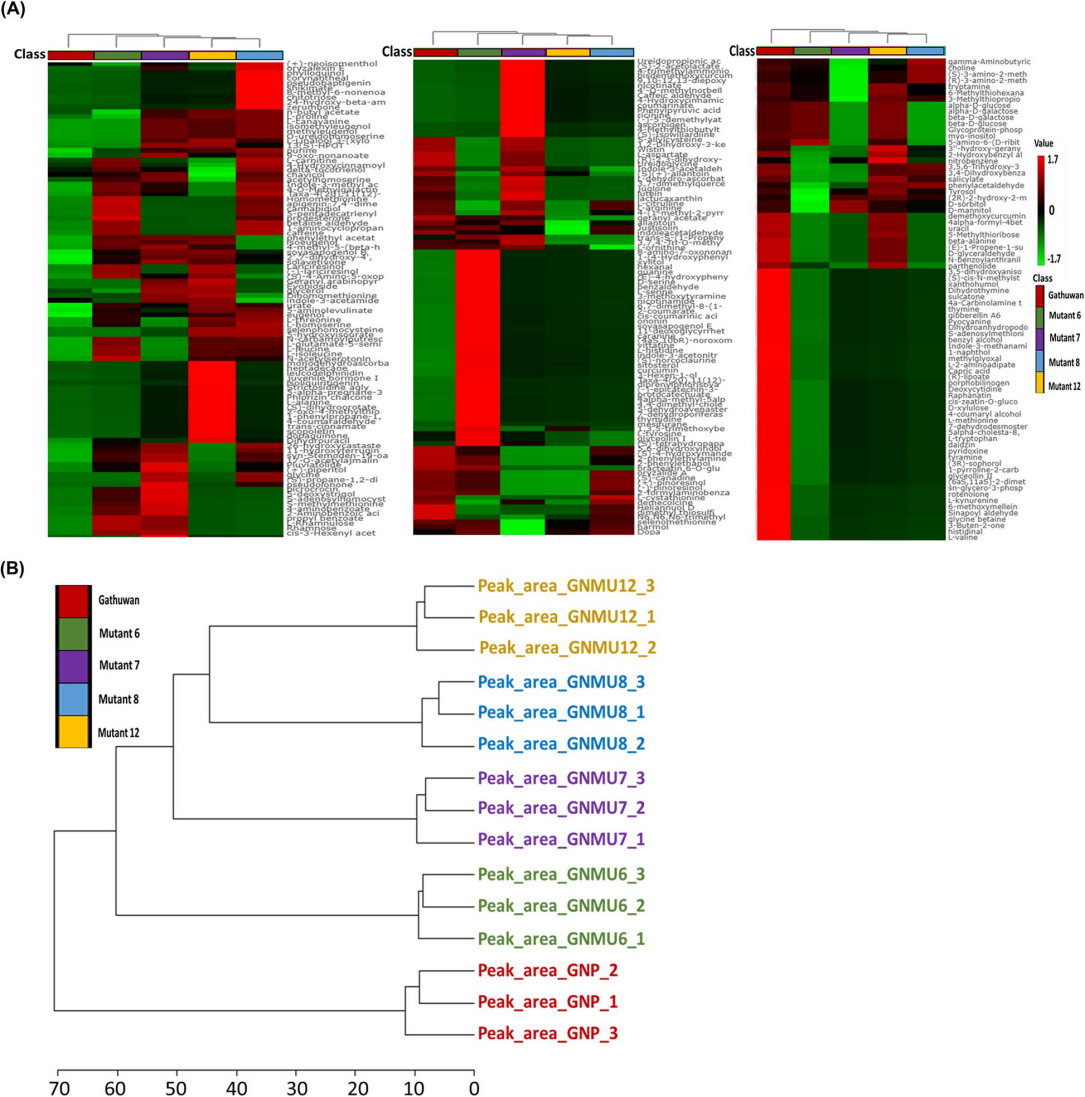
Cluster analysis to visualize the metabolic proximity between Gathuwan and its mutants. **A** Heatmaps showing expression profiles of all metabolites across the samples. The indicator bar on the left side of each heatmap shows the expression level marked by colour intensity. (For convenience, the heatmap has been fragmented into three parts) and (**B**) Dendrogram built on the basis on clustering of samples based on overall pattern of expression

Principal Component Analysis was used to reduce dimensionality of the data. The cumulative score of first two principal components (PCs) was more than 80 percent in parent-mutant 6, parent-mutant 7 and parent-mutant 8 pairs, of which more than 70% variance was accountable to PC1 (S2.3, Supp. File S1). Even with supervised partial least square difference analysis (PLS-DA), the cumulative effects of two components were similar, with variance explained by component 1 remaining same (Fig. 5 A-D). In case of parent-mutant 12 pair, PC1 could explain 67.2% of the variance. The distribution areas of replications of same rice sample agglomerated in the same group without overlapping with that of other sample. A distinct grouping of mutant sample triplicates from that of parent indicated that the former differed significantly from the parent. Loadings plot (Fig. S4 A-D, Supp. Figures) shows the metabolic interrelationships and contribution to the two principal components. Figure 6A-D shows top 40 important features identified using variable importance in the projection (VIP) scores of PC1. Heatmaps (Fig. 7A) showing expression profiles of metabolites across parent and mutants indicated 44 metabolites are downregulated in all the mutants, which were mainly concerned with amino acid metabolism. In cluster analysis, mutant 6 was found to be most similar to the parent than any other mutant (Fig. 7B). Triplicates of all samples showed good correlation amongst themselves and correlation among features led to distinct clusters of metabolites (Fig. S5 A-B, Supp. Figures).

#### Phytochemical composition analysis

**Fig. 8:**
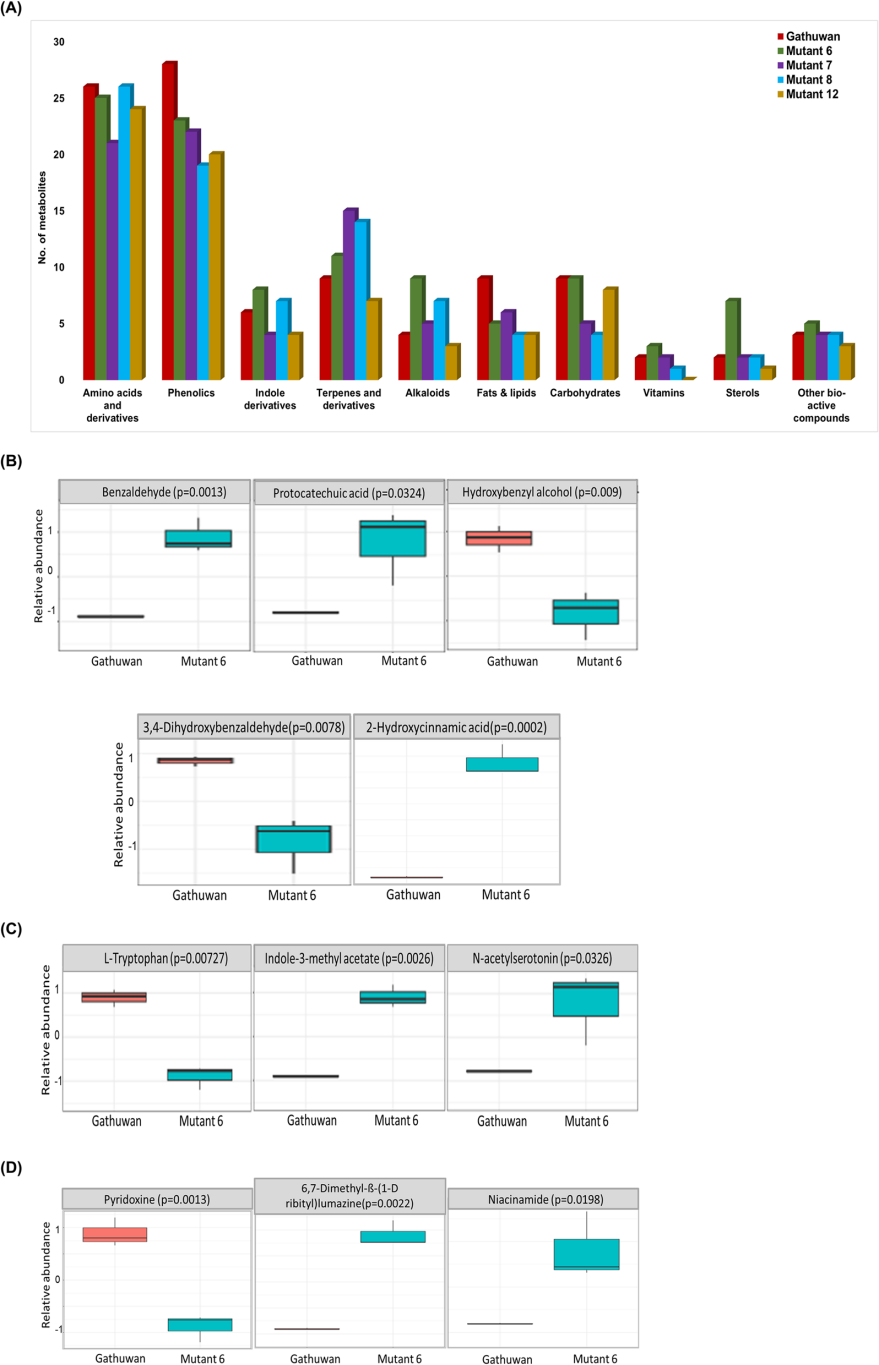
Changes in metabolic profiles among mutants (relative to the parent) in relation to health effects. **A** Bar graph shows number of metabolites belonging to primary and secondary metabolite groups in different samples. **B**-**D** Box plots showing changes in the profiles of bio-active metabolic sets in mutant 6, relative to the parent. **B** Hydroxybenzaldehydes and Hydroxycinnamic acids, (**C**) Indoles and (**D**) Vitamin B complex components

The trend in changes in the pools of secondary and primary metabolites in mutants were compared relative to the parent. More variability was observed in the number of metabolites belonging to different groups of secondary than primary metabolites. Number of terpenes and alkaloidal compounds were found to be increased in majority of mutants, while a decrease was observed in lipid compounds. Pools of amino acids (including proteinogenic and non-proteinogenic) were relatively more uniform in terms of number of metabolites, compared to any other group (Fig. 8A). However, the proportion and metabolism was different, as indicated by pathway analysis. The top 25 metabolic sets altered in mutants with respect to the parent and expression profiles of major groups of natural products have been shown in Fig. S6 A-D and Fig. S7 A-D respectively. Among all mutants, mutant 6 had similar pools of majority of compound classes including amino acid, carbohydrate and vitamin contents and increased pools of terpenes, alkaloids and sterols, relative to the parent. Therefore, the expression pattern of parental bio-active metabolite sets was further explored in mutant 6 (Fig. 8 B-D & S2.4 of Supp. file S1). With respect to the hydroxybenzaldehydes set of compounds, relative content of benzaldehyde and protocatechuic acid was higher in mutant 6 compared to the parent, while the content of hydroxybenzyl alcohol and dihydroxybenzaldehyde was lower. This suggested an increased reduction and reduced hydroxylation of phenolic compounds in the mutant. Compounds including Indole-methyl acetate, Indole-3-acetonitrile and N-acetylserotonin were enriched indoles among mutant 6. Their higher content may be a result of more conversion of tryptophan into indoles. Pyridoxamine (Vitamin B6 forms) levels were downregulated in mutant 6, as was the pathway of its synthesis. However, 6,7-dimethyl-8-(1-D-ribityl)lumazine, a precursor of riboflavin, and niacinamide (Vitamin B3), were enriched instead. Thus, regarding the expression profiles of bioactive compound classes, mutant 6 is expected to retain its functional integrity.

#### Profiles of Plant growth regulators (PGRs) in relation to the plant height

**Fig. 9:**
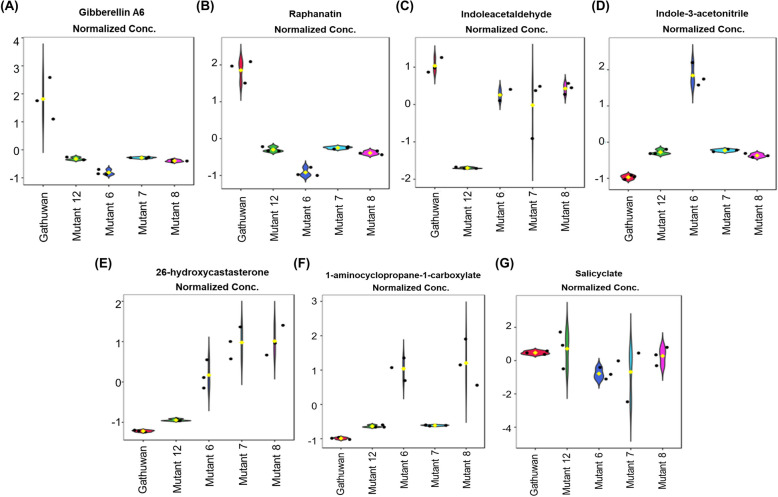
Changes in phytohormonal profiles among mutants (relative to the parent). Violin plots showing changes in contents of phytohormones or their precursors. Violin plot for (**A**) Gibberellin A6, (**B**) Raphanatin, (**C**-**D**) two precursors of auxin, viz. **C** Indoleacetaldehyde and (**D**) Indoleacetonitrile, (**E**) Brassinosteroid, (**F**) Salicylate and (**G**) Precursor for Ethylene synthesis

In the present study, metabolites belonging to different classes of PGRs, viz., Cytokinin (trans-zeatin-7-O-glucoside or Raphanatin), Gibberellin (Gibberellin A6), Brassinosteroid (26-hydroxycastasterone), Salicylic acid (SA)/Salicylate), indole precursors of auxin biosynthesis and precursor for ethylene biosynthesis (1-aminocyclopropane-1-carboxylate), were also found among the differential metabolites (Fig. 9A-G). Gibberellin, the major phytohormone regulating germination and plant height, was downregulated among all mutants, compared to the parent. Cytokinin, another classical plant hormone related to regulation of plant height, could not be observed among mutants, within the detection limits of the instrument. Indoleacetaldehyde and Indole-3-acetonitrile are immediate precursors of Indole acetic acid synthesis in parallel pathways [33]. Both of these were detected in mutant 6, compared to presence of only the former one in the parent. SA was lower in all mutants compared to the parent, whereas brassinosteroid content was higher in majority of the mutants. Salicyclic acid levels governs plant resistance to pests but since the link between the hormonal level in seed and seedling is not distinctive, it cannot predict the mutant responses against pests. The immediate precursor of ethylene biosynthesis, 1-aminocyclopropane-1-carboxylate, was detectable in mutant 6 and mutant 8 only.

## Discussion

Gamma ray mutagenesis has been widely used to generate genetic variation in crop plants, including rice. The present study demonstrate that gamma irradiation can be effectively utilized to generate rice genotypes with improved agronomic performance while retaining or enhancing the key metabolites associated with health benefits of the parent rice variety. In this study, four mutant lines were selected based on their stable expression of reduced plant height and maturity duration traits, from M3-M6 generations. Two of these mutants (mutant 6 and mutant 7) exhibited higher yield than the parent variety. In view of the limited research on the metabolic effects of gamma radiation in genetically stabilized mutant population, we employed global metabolomics to evaluate stable gamma-induced rice mutants in comparison to the parent line, with the objective to identify lines that combine agronomic advantage with potential nutraceutical value. We observed a heterogenous pattern of metabolic changes among all four Gathuwan mutants, despite their relatively similar phenotype. Whether this is attributable to random and dispersed small genetic changes caused by the radiation or a consequence of interaction with matter led metabolic changes inherited further, is not clear.

Global metabolomics revealed that, as compared to the parent, Vitamin B6 pathways was significantly affected among mutants and most of the downregulations were observed in metabolites pertaining to amino acid metabolism. Notably, the metabolism of arginine and branched-chain amino acids did not show significant alterations, unlike their synthesis. Therefore, these pathways do not provide substantial insights into the functional potency of the mutants compared to the parent. Further, phenylalanine metabolism was among the enriched pathway in all samples. The observed differences in content of phenolics such flavonoids, isoflavanoids, lignins, diarylhepatnoids implied changes in the relative importance of pathway nodes diverging from phenylalanine metabolism. Notably, mutant 6 expressed compounds from key functional phytochemical classes (e.g., alkaloids, sterols, and terpenes) that were absent in the parent line, while also minimizing the loss of existing metabolites. This makes mutant 6 a promising candidate for selection in medicinal applications. Compounds belonging to phenolics group were least affected in mutant 6. Although several metabolite groups such as indoles, hydroxybenzaldehydes, hydroxycinnamic acids and pyridoxamines, which have been previously linked to the functional roles of Gathuwan brown rice [31], were altered in mutant 6, they appeared to be chemically modified to potent molecules with similar activity and mechanisms. This study, therefore, provides an integrated evaluation of the balance between biosynthetic and metabolic pathways, with a focus on the changes in overall metabolite pools rather than individual compounds. It also emphasizes on the functional relevance of these metabolic changes to identify the most promising candidate mutant.

Further, we observed that classical phytohormones, GA, CK, IAA and ethylene were generally downregulated in mutants. Since rapid and uniform germination has been linked to variation in plant height differences [34], it is plausible that plant height is influenced by the distribution of these phytohormones in different parts of the rice seed. They regulate the seed germination and have a decisive impact on plant height. Among them, GA and CK, are key regulators of germination [35, 36]. In our study, reduced levels of gibberellins and zeatin in rice grain of mutants correlated with reduction in plant height. This hormone interaction is further supported by the known involvement of GA and CK crosstalk in regulating plant height through the OsNAC103 transcription factor [37]. Although brassinosteroids-GA interaction is also known to regulate plant height [38], brassinosteroid levels in this case, may either not have a role in plant height regulation or the levels observed in mutant 6, 7 and 8 are high enough to exert suppressive effects on GA biosynthesis. Notably, mutant 6 contains immediate precursors of both IAA biosynthesis and ethylene biosynthesis, suggestion it may have distinct developmental pattern than the parent and other mutants. Higher spikelet fertility, a trait affecting the yield, has been associated with enclosed stigma under high heat conditions and GA application antagonizes the process by promoting exsertion of stigma [39]. Further, Ethylene affects grain filling process by affecting amino acid biosynthesis [40]. mutant 6, with reduced gibberellin and higher spikelet fertility, and higher ethylene content might be conforming to similar mechanism of increased yield under high heat conditions and thus, provides yet another prospect for which the mutant can be evaluated.

Besides causing a wide range of mutations, Gamma rays may also trigger random epigenetic changes, particularly demethylation, which may significantly influence gene expression [41]. Increasing evidence shows that epigenetic regulation, through DNA methylation, histone modifications, and small RNAs, plays a critical role in controlling plant metabolic networks, especially those governing stress responses and secondary metabolism [42, 43]. These modifications influence gene expression both locally and distantly, shaping the plant’s developmental and physiological outcomes and may exhibit transgenerational inheritance [43, 44]. Thus, the metabolic shifts observed among mutants may not be solely attributed to genetic mutations but could also reflect underlying epigenetic modifications induced by gamma irradiation. Future studies integrating epigenomic profiling will be essential to uncover the specific methylation dynamics associated with key metabolic traits. This will not only enhance our understanding of radiation-induced variation but also support the use of epigenetic diversity as a valuable tool in crop improvement, offering new avenues for trait enhancement without genetic erosion. The study, in addition to highlighting the potential changes in the biological activity of mutants and predicting their development and physiological responses to stress, also raises interesting possibilities to explore.

## Conclusion

In this study, metabolic profiles of agronomically superior mutants in the genetic background of Gathuwan, were analysed to assess their biochemical similarity to the parent. Among the mutants, mutant 6—characterized by reduced plant height, early maturity, and higher yield—retained the highest percentage of parental metabolites and exhibited the most significant upregulation of features relative to the parent. The pool size of functional metabolite sets was also better preserved in mutant 6 than other mutants. Additionally, the bioactive metabolite sets and their functionality in the parent variety were largely preserved, despite a few modifications. Based on these findings, mutant 6 was identified as the closest to the parent in terms of phytochemical composition and as a promising candidate with respect to both agronomic and biochemical traits. The study demonstrates the potential of biochemical screening to aid in the selection of rice mutants with health-promoting properties. The integrative data provide a predictive basis for identifying the most promising candidate, in terms of bioactivity and agronomic performance. However, bioactivity testing and targeted quantification of metabolites are needed to validate these predictions.

## Supplementary Information

Supplementary Material 1.

Supplementary Material 2.

## Data Availability

All data generated or analysed during this study are included in this manuscript and its supplementary information files.

## Abbreviations

ANOVA: Analysis of Variance
CK: Cytokinins
ERK: Extracellular signal-Regulated Kinases
FC: Fold Change
FDR: False Discovery Rate
GA: Gibberellic acid
GABA: Gamma Amino Butyric Acid
KEGG: Kyoto Encyclopedia of Genes and Genomes
IAA: Indole Acetic Acid
LC–MS/MS: Liquid Chromatography-Tandem Mass Spectrometry
Nrf2: Nuclear factor erythroid 2-related factor 2
PCA: Principal Component Analysis
PLS-DA: Partial Least Square-Discriminant Analysis
VIP: Variable importance in projection
