# Supplementary material for: Agronomic improvement using gamma ray induced mutagenesis is associated with changes in phytochemical and phytohormonal profiles in functional rice variety ‘Gathuwan’
Source: BMC Plant Biol. 2025 Aug 12;25:1069. doi: 10.1186/s12870-025-07036-1 (PMC12341213; doi:10.1186/s12870-025-07036-1)
Supplement: Supplementary file 1 — Supplementary Material 1. [file 12870_2025_7036_MOESM1_ESM.docx]

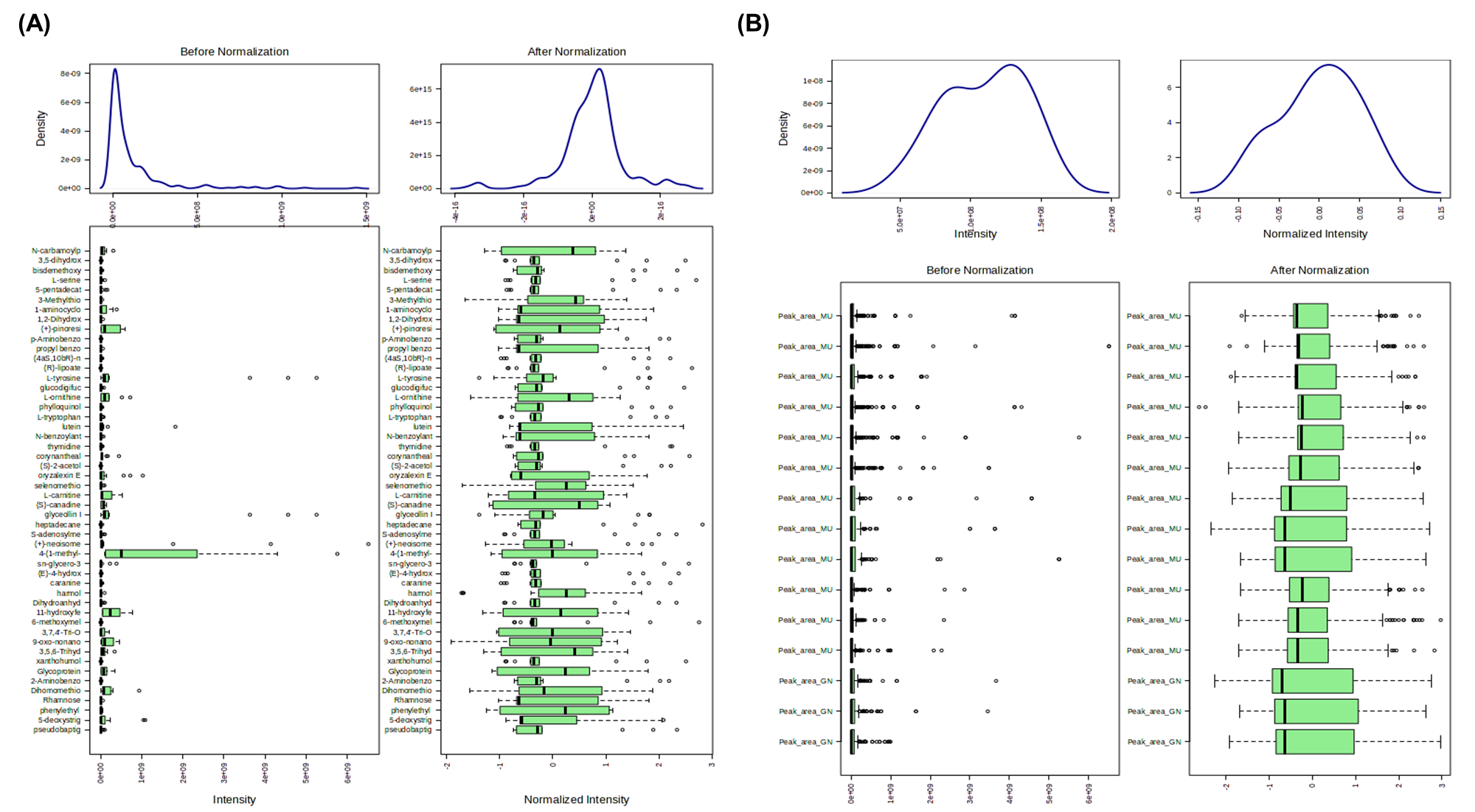


**Fig. S1- Feature and Sample data normalization respectively for pair-wise metabolomic comparison of all mutants compared to parent.**


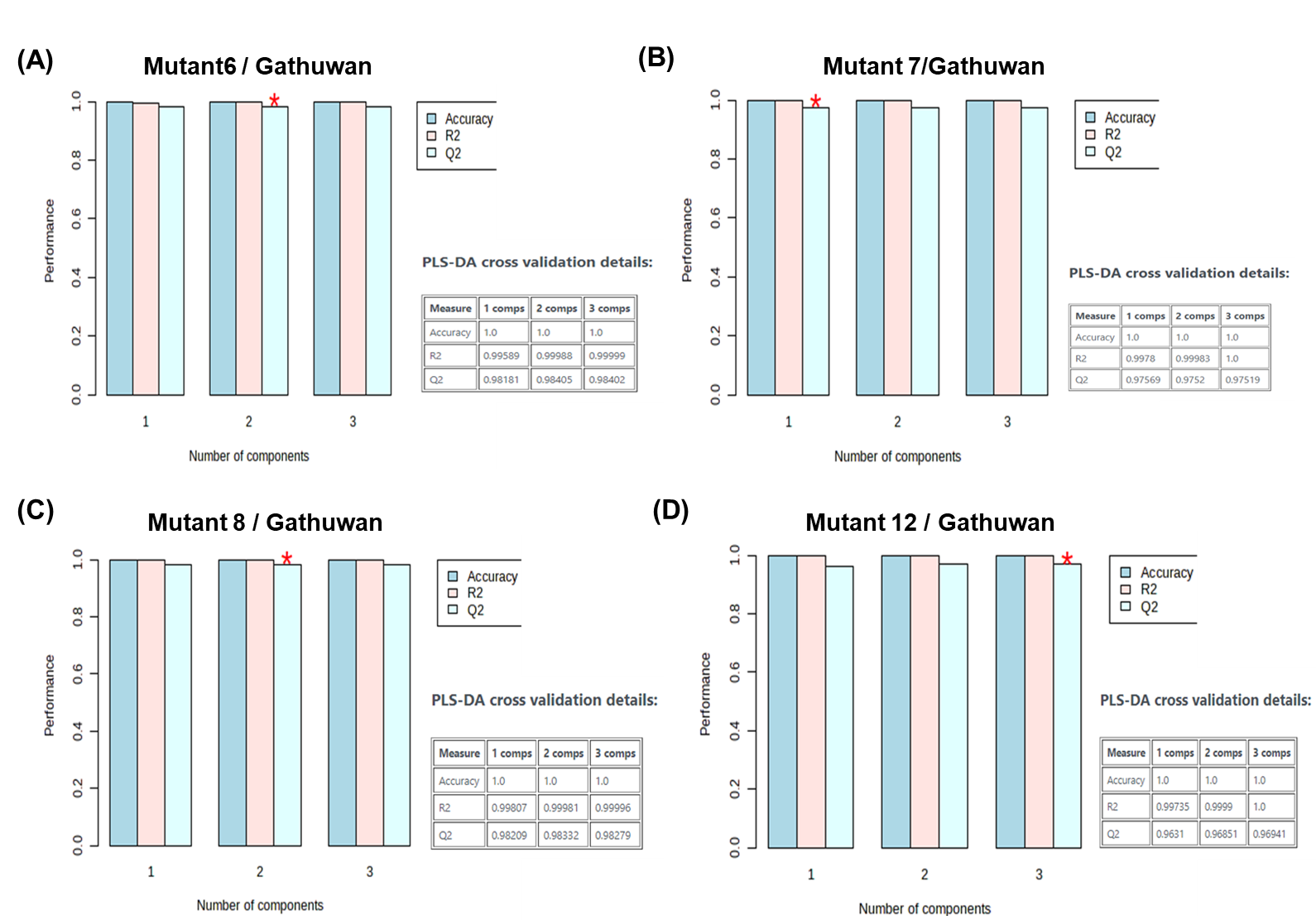


**Fig. S2. Partial least squares-discriminant analysis (PLS-DA)-cross-validation plots, along with respective details, indicating quality and predictability of the statistical test for pairwise comparison of (A) Mutant 6, (B) Mutant 7, (C) Mutant 8 and (D) Mutant 12 with Gathuwan (Parent).**


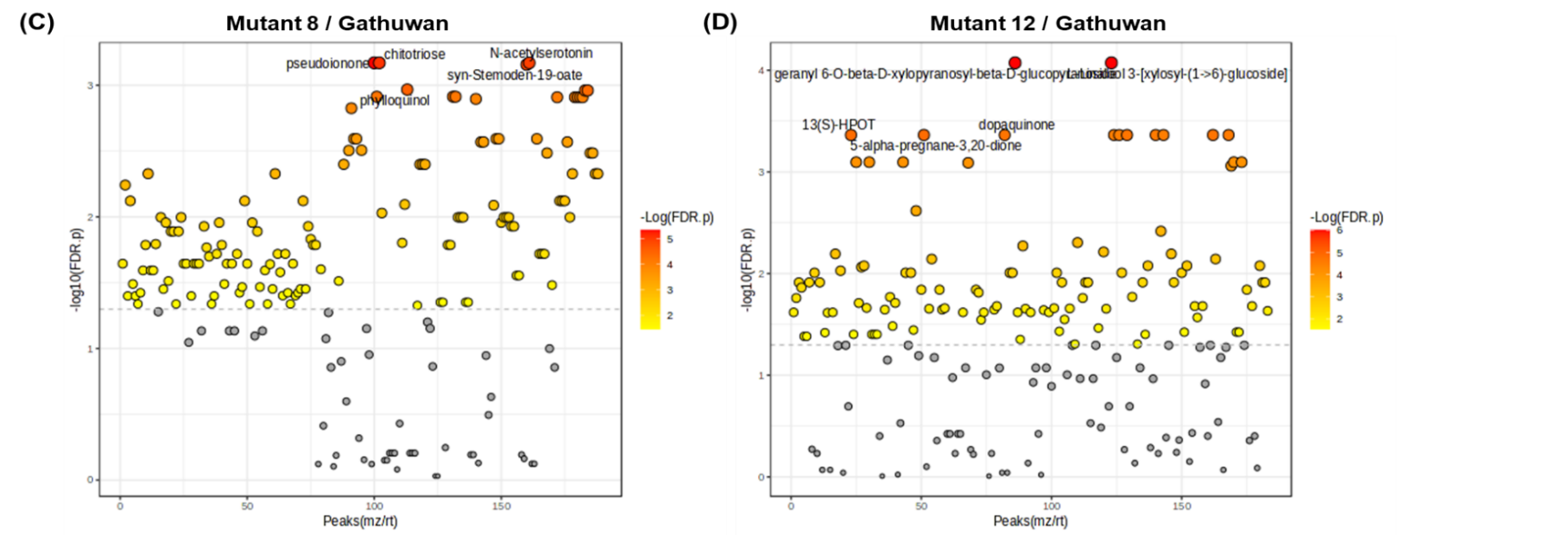

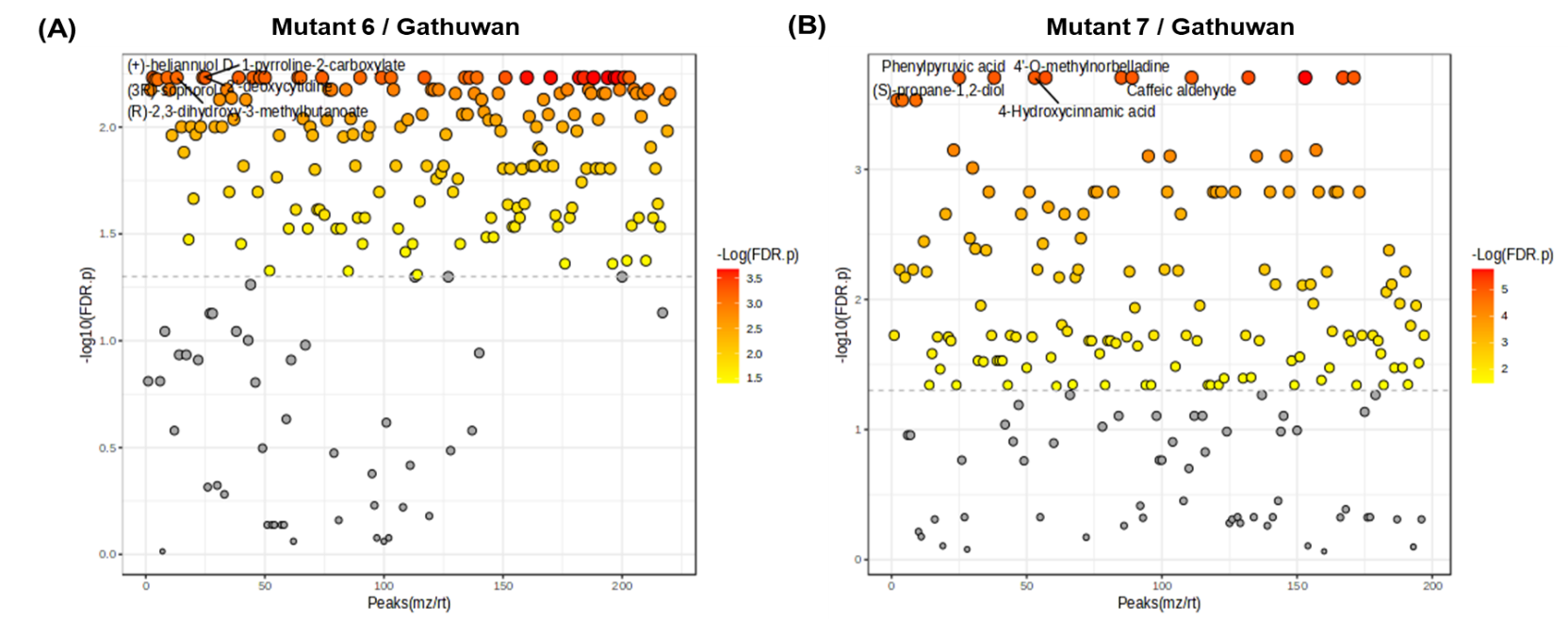


**Fig. S3. Univariate analysis (t-test) to identify significantly altered features between (A) Mutant 6 and Parent, (B) Mutant 7 and Parent, (C) Mutant 8 and Parent and (D) Mutant 12 and Parent**


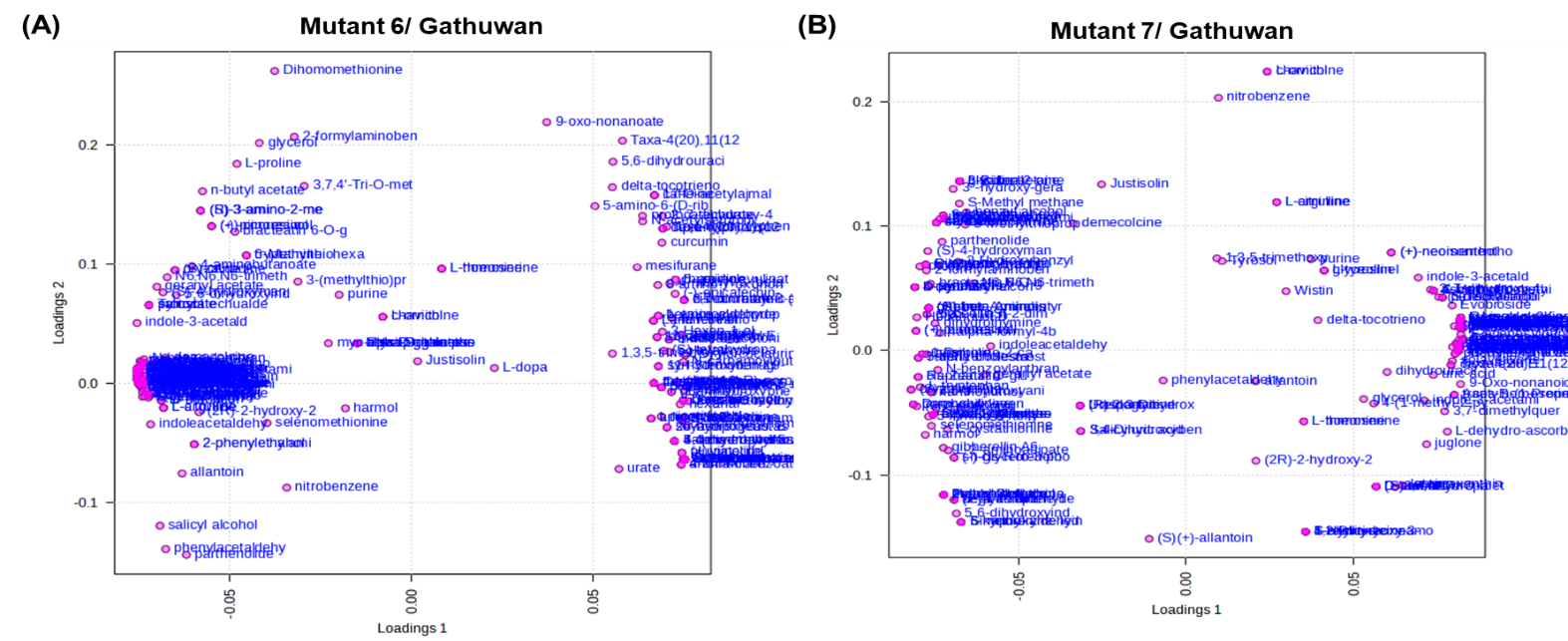


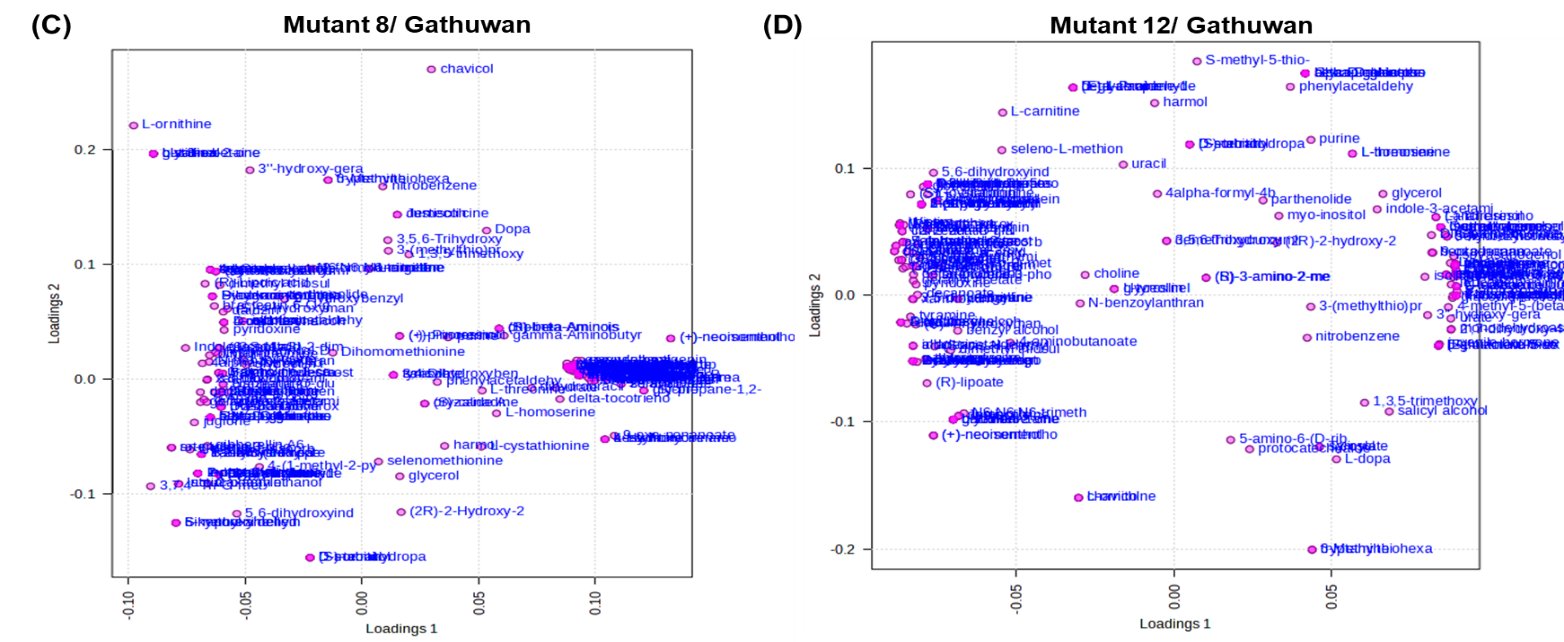


**Fig. S4.** **Partial least squares-discriminant analysis (PLS-DA) based loadings plot indicating contributions of metabolites to two principal components in pair-wise comparison of (A)Parent-Mutant 6, (B) Parent-Mutant 7, (C) Parent-Mutant 8 and (D) Parent-Mutant 12**


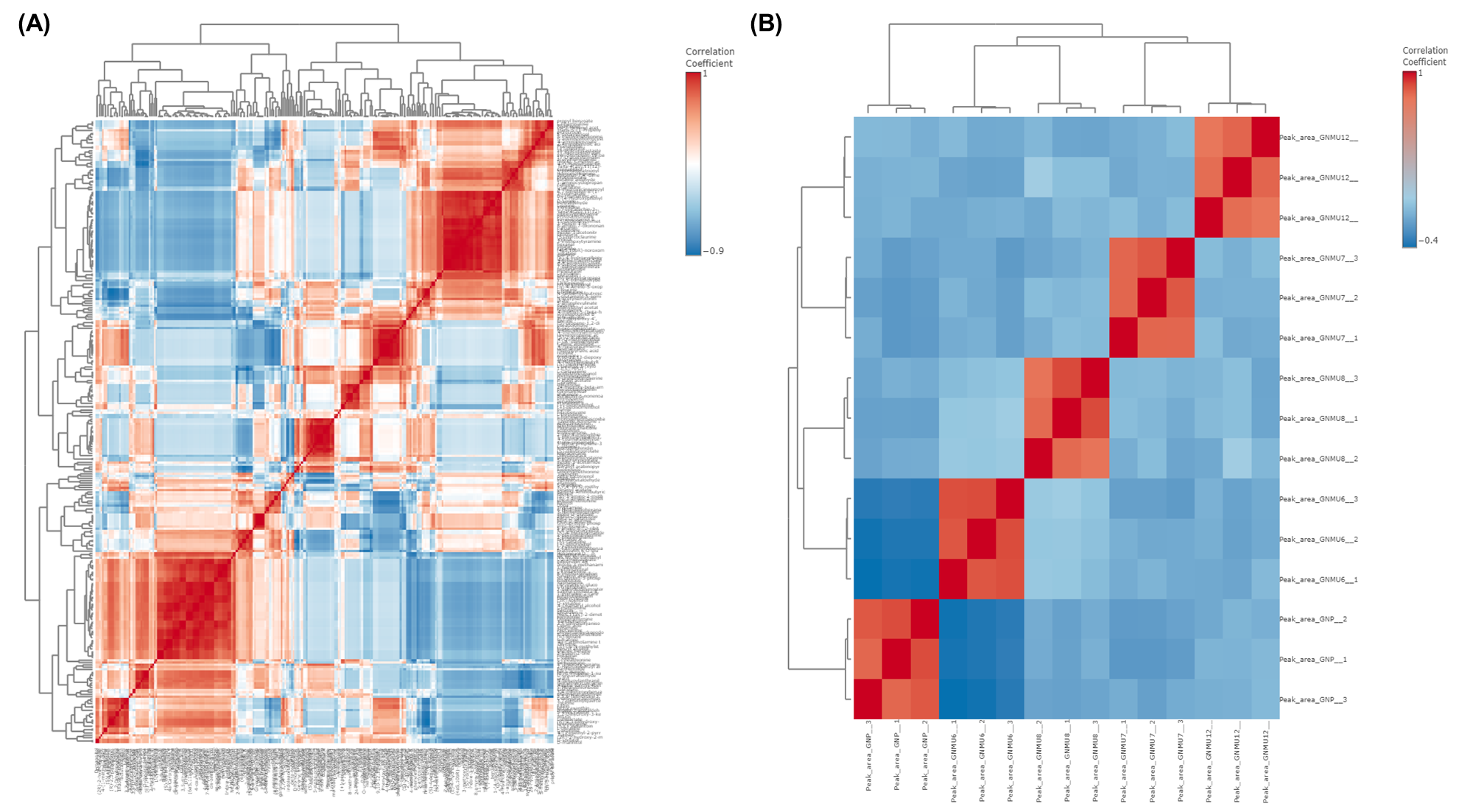


**Fig. S5. Plots showing correlation among (A) Features and (B) Samples**


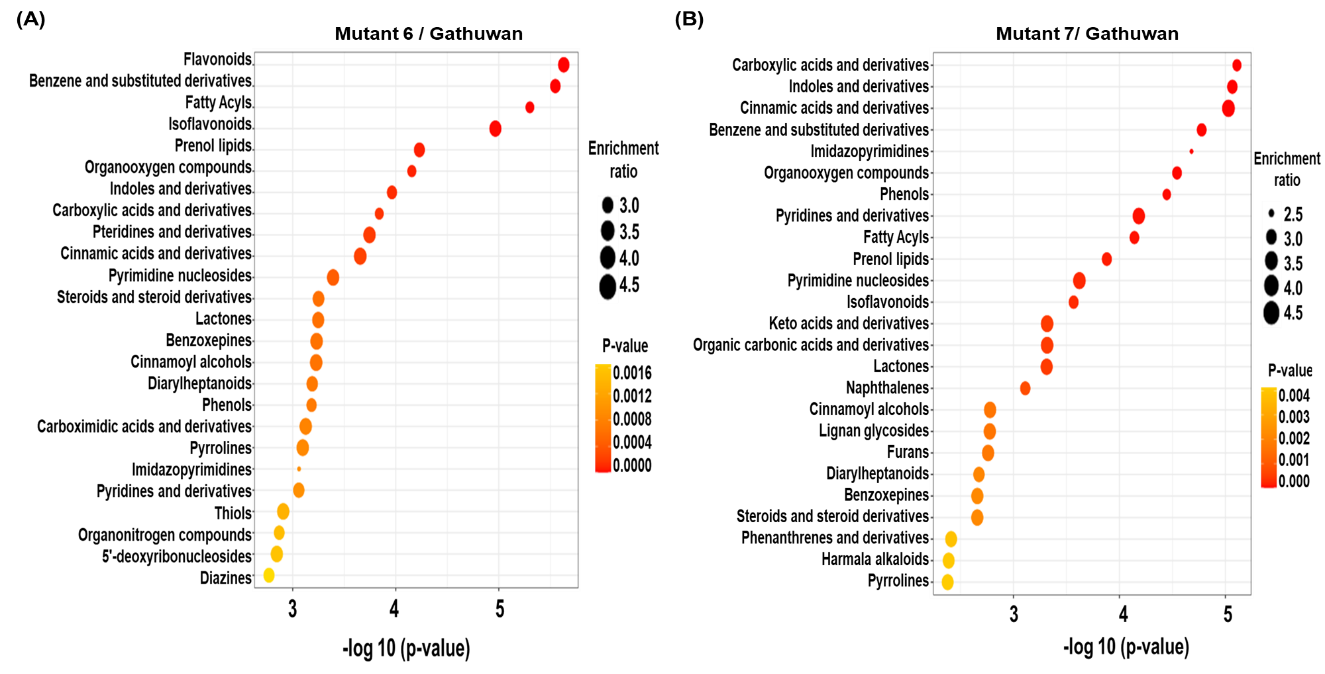

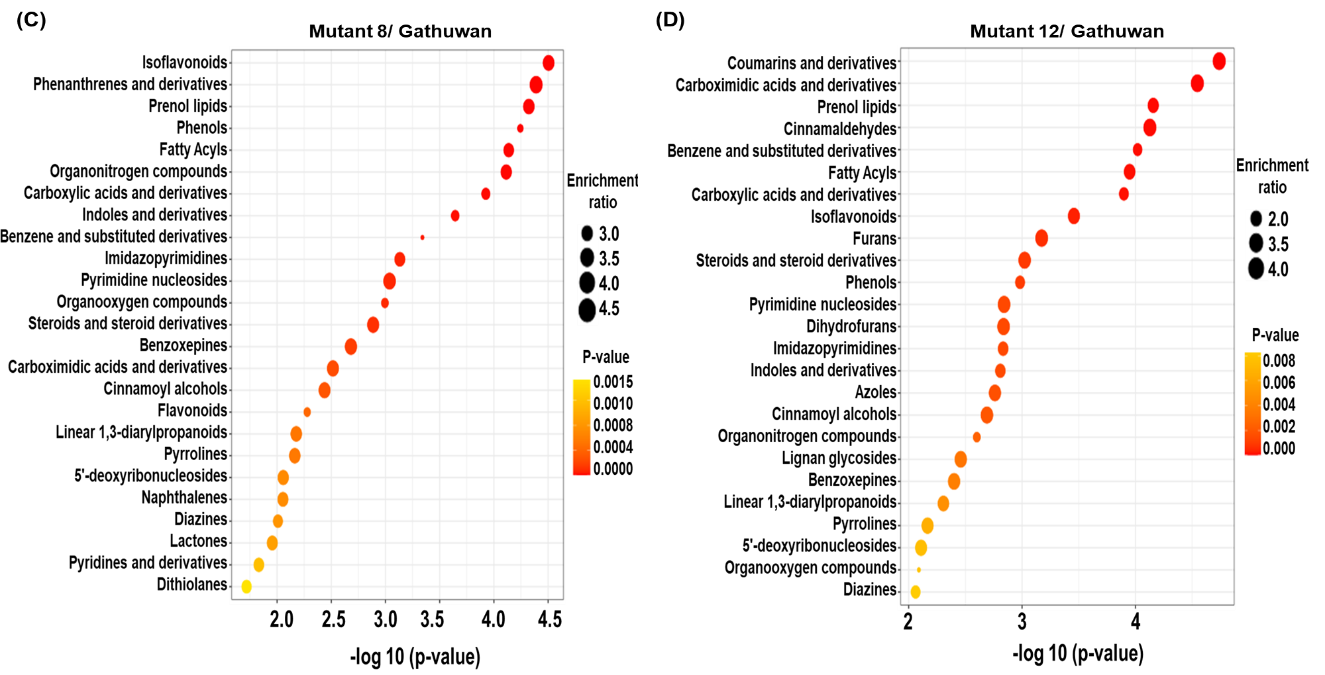


**Fig. S6. Enrichment analysis of metabolite sets in pairwise comparison of (A) parent-mutant 6, (B) parent-mutant 7, (C) parent-mutant 8 and (D) parent-mutant 12 respectively.**


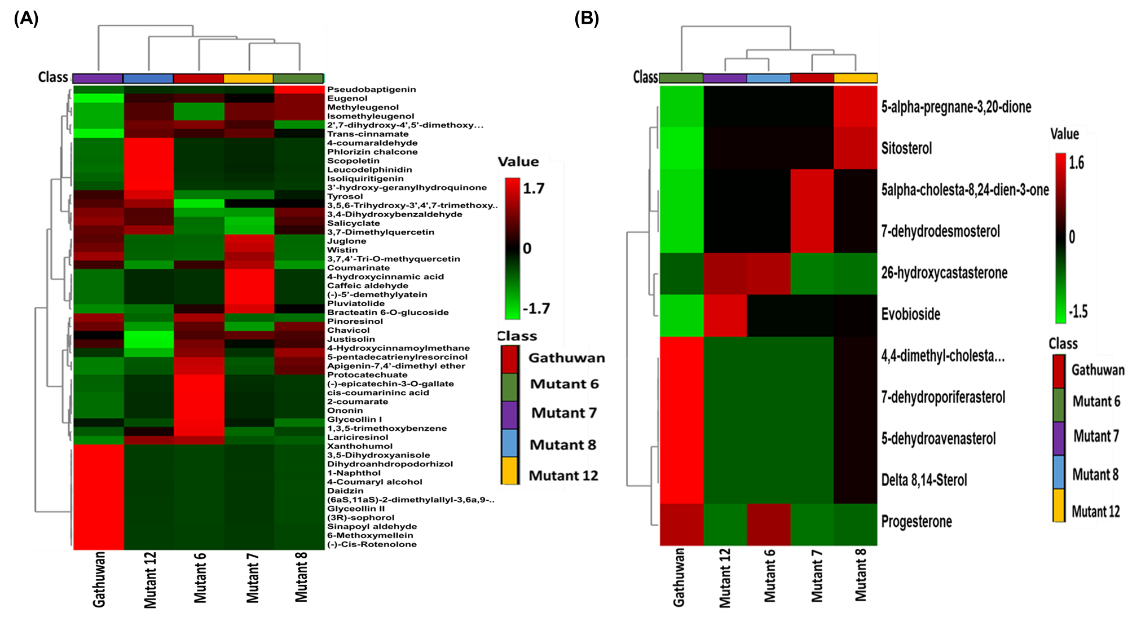


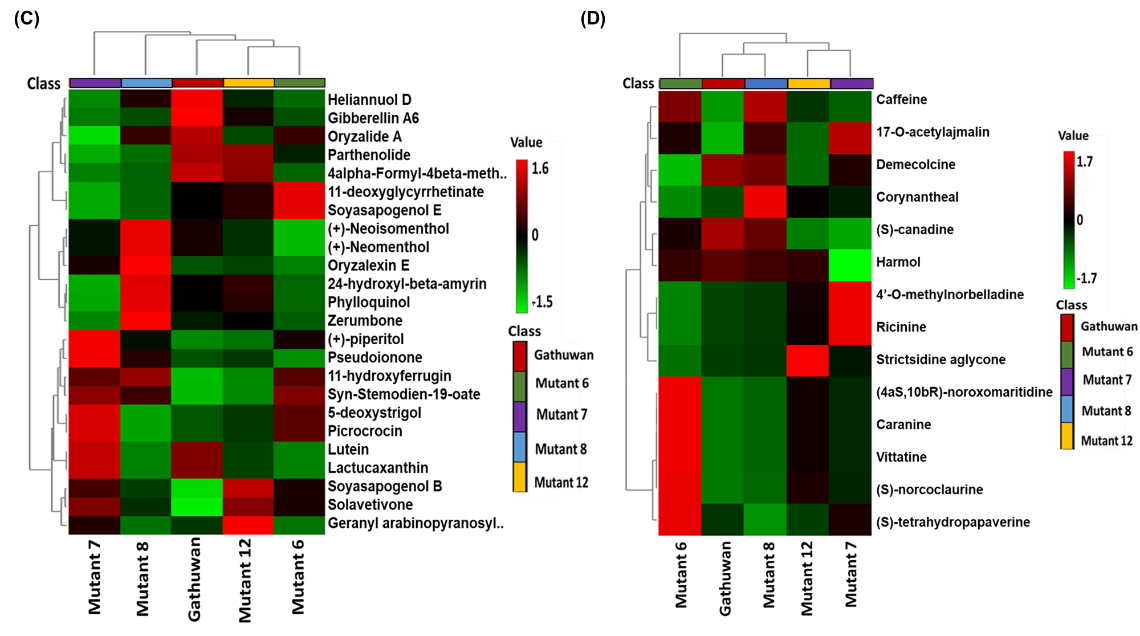


**Fig. S7. Heatmaps showing expression profiles of (A) Phenolic compunds, (B)Sterols, (C)Terpenes and (D) Alkaloids, among the studied samples.**

**Supplementary File 1**

**S2.1 Metabolite distribution data. (A) Distribution of total metabolites into various compound classes and (B) Proportion of of functional metabolites in Gathuwan and its mutants.**

**S2.2 Univariate analysis for statistical significance of features (A) Fold change analysis (B) t-test for significance and (C) Volcano plot dats**

**S2.3 Multivariate analyses to find out metabolic diversity/proximity between Gathuwan and mutants (A) Principal Component analysis and (B)** **Pair-wise PERMANOVA Results.**

**S2.4 Presence of bio-active compounds in Mutant 6 relative to the parent.**
